# Research on Fault Prediction of Power Devices in Rod Control Power Cabinets Based on BiTCN-Attention Transfer Learning Model

**DOI:** 10.3390/mi15111326

**Published:** 2024-10-30

**Authors:** Zhi Chen, Liqi Ye, Yifan Jian, Meiyuan Chen, Yuan Min

**Affiliations:** 1 National Key Laboratory of Nuclear Reactor Technology, Nuclear Power Institute of China, Chengdu 610213, Chinacmy_work@sina.cn (M.C.); npic_my@163.com (Y.M.); 2 School of Computer Science, University of South China, Hengyang 421200, China

**Keywords:** bidirectional temporal convolutional attention network, transfer learning, power device, fault prediction

## Abstract

The Insulated Gate Bipolar Transistor (IGBT) is the key power device in the rod control power cabinet of nuclear power plants; its reliable operation is of great significance for ensuring the safe and economical operation of the nuclear power plants. Therefore, it is necessary to conduct fault prediction research on IGBT to achieve better condition-based maintenance and improve its operational reliability. However, power cabinets often operate under multiple, complex working conditions, so predicting IGBT faults from single working condition data usually has limitations and low accuracy. Its failure probability has an important relationship with the actual operating conditions of the cabinet. In order to improve the reliability and maintainability of the control power cabinet in nuclear power plants, this paper takes IGBTs in the rod control power cabinet as the object and makes full use of the data of IGBTs under multiple working conditions to carry out research on the cross-condition fault prediction of IGBTs under multiple-source working conditions. A transfer learning (TL) model based on a bidirectional time convolutional network (BiTCN) combined with attention was proposed to solve the problem of low accuracy of cross-operating fault prediction in a multi-source domain. Firstly, an IGBT fault simulation model was built to collect the life cycle state data of the module under different working conditions. Then, after pre-processing such as removing outliers, kernel principal component analysis (KPCA) was used to integrate all source domain data, obtain source domain characterization data, and train the BiTCN-attention model. Finally, the BiTCN-attention model trained in the source domain was transferred, and the model was fine-tuned according to the target domain data. Simulation results show that the accuracy of the proposed BiTCN-attention transfer learning prediction method can reach more than 99%, which is significantly better than that of the recurrent neural network transfer learning (RNN-TL) model, long short-term memory network transfer learning (LSTM-TL) model, gated cyclic unit transfer learning (GRU-TL) model, and time convolutional network transfer learning (TCN-TL) model. This method can not only reduce the inconsistency of fault characteristic values caused by changes in working conditions but also accurately predict the degradation trend when only early fault data are available, providing an effective solution for IGBT fault prediction across working conditions in multi-source domains.

## 1. Introduction

The rod control system is responsible for the electrical control of the control rod drive mechanism and plays an important role in nuclear power plants. The rod control power cabinet is a crucial device in the system. Its function is to provide the corresponding sequential current for the three coils of the driving mechanism so as to control the rise and fall of the control rods to adjust the power of the reactor [1]. Its normal operation plays an important role in the safe and economic operation of the nuclear power plant. However, in bad working environments and under different working conditions, the Insulated Gate Bipolar Transistor (IGBT), which is the key power device in the rod control power cabinet, often appears in all sorts of performance degradation states, such as bridge arm straight, too much current growth rates, overload, and poor heat dissipation. In severe cases, it can even lead to IGBT burning [2,3]. These failures of IGBTs often further lead to the failure of the rod control power cabinet, causing an unplanned shutdown of the nuclear power plant, adding a lot of additional time costs, labor costs, and resource costs, resulting in huge economic losses. Therefore, it is of great significance to study the fault prediction method of IGBT in the rod control power cabinet for improving the operational reliability and economy of nuclear power plants.

Until now, there have been many research achievements in the related industrial fields for fault prediction of key equipment or components [4,5,6,7]. In recent years, deep learning-based methods have received increasing attention; the method has achieved some research results in IGBT fault prediction under a single working condition. For example, Jiang Chuang et al. proposed a deep learning method with multidimensional time-domain features and attention mechanism for predicting IGBT modules [8]; Chen Zhengxiong et al. proposed an IGBT aging prediction method based on the Improved Whale Optimization Algorithm (IWOA) to optimize Support Vector Regression (SVR) [9]. However, in practical engineering applications, the IGBT fault prediction model pre-trained by a single operating condition data set can only make a good judgment on the data under such operating conditions. When IGBT test samples are collected under different operating conditions, their generalization ability is insufficient, resulting in a decline in the prediction ability [10,11]. In actual operation, the rod control power cabinet will also face different operating conditions, so the performance degradation trend of IGBT is closely related to the operating conditions. The existence of different working conditions poses challenges to IGBT fault prediction based on deep learning: the IGBT fault prediction method based on deep learning is dependent on training data, and various parameters of IGBT modules vary greatly across working conditions, resulting in poor model adaptability.

At present, the mainstream method of IGBT fault prediction under cross-operating conditions is the transfer learning (TL) algorithm. This algorithm achieves fault prediction across working conditions by reducing the distribution difference between the source and target domains. For example, Zhong Zhiwei et al. proposed a prediction method of transfer learning based on probabilistic sparse self-attention mechanism and domain adaptive principle [12]; similarly, Deng Wujin et al. constructed a transfer learning method of Markov mode transition stochastic process to transfer knowledge from the source domain (DC voltage gate biased IGBT module) to the target domain (Pulse Width Modulation (PWM)-controlled IGBT module) so as to improve the estimation accuracy of the mode transfer probability of the PWM-controlled IGBT module and achieve fault prediction with a small amount of target domain data [13]. Although the above studies greatly expand the scope of the application of prediction models and achieve cross-condition learning, they mainly learn from a single source domain, and the lack of diversity of data learned from a single source domain may cause the model to fail to capture comprehensive features, increasing the risk of overfitting. This characteristic of depending on specific working conditions makes the model inadaptable, which limits its effective application in complex and dynamic environments. Therefore, in order to improve the robustness and portability of the model, it is particularly important to integrate data from multiple source domains.

Aiming to solve the above problem of low accuracy of cross-operating fault prediction by obtaining characterization data from a single source domain, this paper proposes a time series model and transfer learning method combining multi-condition data to solve the problem of IGBT fault prediction under cross-condition. The main innovations of this method include: (1) Considering that source domain data in practical applications may come from multiple operating conditions, Kernel Principal Component Analysis (KPCA) is used to integrate the available knowledge of all source domains to predict the target operating conditions. (2) It adopts Bi-directional Temporal Convolutional Network (BiTCN) [14], considering the past and the future information at the same time, and, combined with the attention mechanism, fully extracts the important characteristics in the time-series data, gives different attention weight to input information of different times, and captures patterns and associations in time-series data more comprehensively. (3) The feature set model trained under different working conditions is transferred to the feature set of the target working conditions in order to reduce the difference of fault feature values caused by the change of working conditions and make fault prediction more accurate.

## 2. Correlation Algorithm

### 2.1. KPCA Principle

Kernel Principal Component Analysis (KPCA) is the application of the kernel function to principal component analysis, which can solve the nonlinear feature extraction problem [15]. First map to a higher-dimensional space, then use linear dimensionality reduction to map to another lower-dimensional space. This means that the original data sample η is transformed into a two-dimensional covariance matrix by the kernel function as follows:

(1) $$\sum =\frac{1}{n}\sum_{i=1}^{n}\varphi (\eta^{i})\varphi {{(\eta }^{i})}^{T}$$

In order to obtain the eigenvectors $U_{b}$ of this covariance matrix, i.e., the principal component matrix of the vectors, we can use the following equation:

(2) $$U_{b}=\frac{1}{n}\sum_{i=1}^{n}\alpha^{i}\Lambda \left(x^{i}\right),\Lambda =\varphi (\eta )\varphi {\left(\eta \right)}^{T}$$

where α can be obtained by extracting the eigenvector of the kernel matrix Λ.

### 2.2. BiTCN-Attention Model

Temporal Convolutional Network (TCN) was put forward in recent years by Bai et al. [16]. According to a study, TCN is more precise, concise, and clear when processing sequence data compared with traditional cyclic networks, such as long short-term memory network (LSTM) and recurrent neural network (RNN). The structure of TCN integrates dilative convolution and causal convolution, which is suitable for time series modeling and can effectively reduce the computation and maintain a large receptive field so as to obtain information in a longer time span.

**Figure 1:**
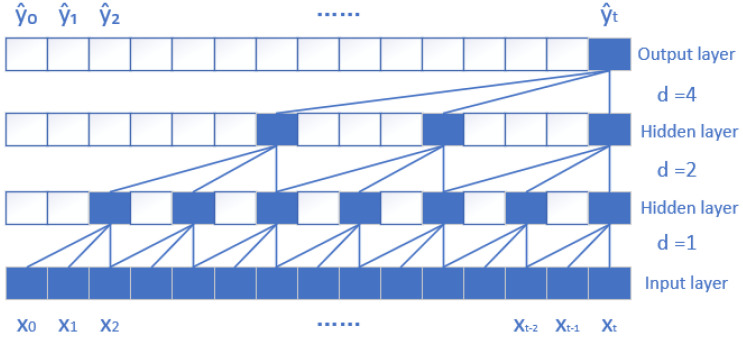
TCN extended causal convolutional structure.

TCN is a one-dimensional convolutional network structure, which mainly consists of extended causal convolution and residual connection, and its structure is relatively simple. The extended causal convolution structure of TCN is shown in Figure 1 [17]. During the convolution operation of TCN, the input data can be sampled at a certain interval in order, and the certain interval is the sampling rate. In the figure, d represents the sampling rate; d = 1 indicates that every point is collected, and the higher the layer, the larger the d is. In the TCN network, the residual connection consists of a residual module that includes dilated causal convolution, weight normalization, activation functions, and regularization. This residual structure can avoid the loss of more information in the process of feature extraction, retain as much information as possible, and improve the accuracy of the model.

**Figure 2:**
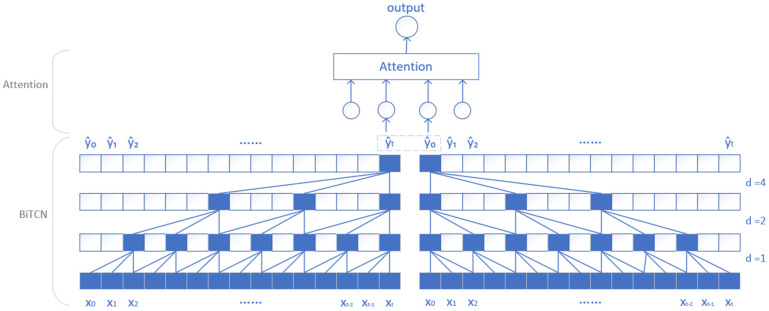
BiTCN-Attention model structure.

BiTCN is an improved version based on TCN. The disadvantage of TCN is that only forward features can be extracted and backward features cannot. To address this issue, BiTCN introduces a bidirectional convolution structure based on TCN, which retains the processing capability of TCN for time series and increases its ability to capture bidirectional information of the sequence. It can consider the past and future of information at the same time, thus enhancing its ability to fully capture the time series data of the patterns and associations. In order to make the model more flexible in learning the importance of different time points in the sequence, more attention needs to be given to important time steps when processing time-series data, further improving the prediction accuracy of the model. Therefore, this article incorporates an attention mechanism into the BiTCN model [18] and adopts the BiTCN-attention model. The structure of this model is shown in Figure 2.

### 2.3. Transfer Learning

Transfer learning is an algorithm that applies previous knowledge to a similar field or task and is also a subset of deep learning. The domain of learning is called the source domain, and the domain to be solved is called the target domain. The source domain corresponds to the original task, and the target domain corresponds to the target task. The transfer learning method can solve the problem of poor cross-condition prediction and save the calculation cost and training cost [19].

Transfer learning is defined as follows: Given a source domain D_S_ = {x_i_,y_i_} (i = 1, 2……n^s^) and a target domain D_T_ = {x_j_,y_j_} (j = 1, 2……N^T^), when the transfer condition is established, the model uses the knowledge learned from the source domain data to approximate a prediction function $f\left(•\right)$ on the target domain so that $f\left(•\right)$ can achieve the maximum prediction accuracy on the target domain. The expression is as follows [20]:

(3) $$f\left(•\right)={\mathrm{arg min}}_{f\left(•\right)}E_{\left(x,y\right)\in D_{T}}l[f\left(x\right),y]$$

where l(•,•) is the prediction error and $f\left(•\right)$ is the prediction function.

## 3. Transfer Learning Model Based on BiTCN-Attention

**Figure 3:**
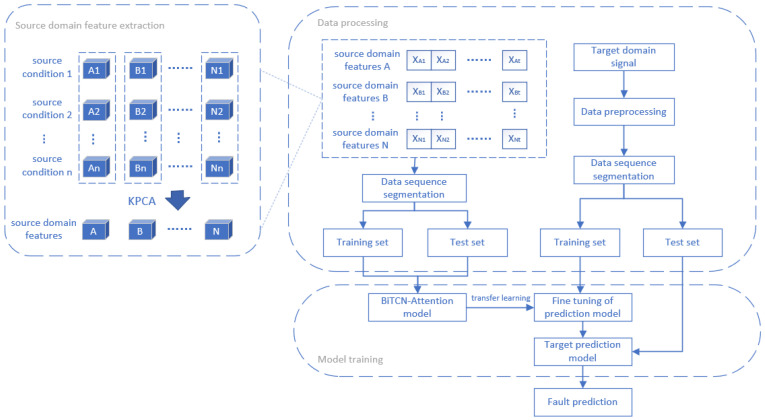
Overall framework of fault prediction.

In this paper, a transfer learning method based on a Bidirectional Temporal Convolutional Network with attention (BiTCN-attention) is proposed, which migrates from the multi-source condition with lifecycle degradation data to the new target condition for fault prediction. The overall framework of fault prediction based on this method is shown in Figure 3.

From Figure 3, the specific implementation steps of the fault prediction method proposed in this paper are as follows:

- Source domain feature extraction: Collect multiple source working condition signals for data pre-processing. The sample length of each source condition is truncated to ensure that the length of each sample is consistent. In order to obtain the available knowledge of all source domains and reduce the model training deviation caused by redundant abnormal data, the KPCA method is used to integrate all source operating signals, extract distinguishing features from different data sources, and obtain the feature data of source domains.
- Data processing: First, the obtained source domain characteristic signal is segmented, and the training set and test set are divided proportionally. Second, after preprocessing the signal of the target domain, it is segmented, and the training set and test set are divided proportionally.
- Model training: First, the model parameters are initialized, and the optimizer is selected to iteratively train the training set. Then, the trained BiTCN-attention model is used to predict the test set, and the corresponding prediction data are generated. Finally, when the prediction model is built in the target domain, the network parameters of the source domain are directly transplanted, and some BiTCN-attention network parameters are fixed and will not be changed again. In the data training of the target domain, another part of the variable network parameters is adjusted to better fit the input–output mapping relationship of the target domain.

## 4. Data Acquisition Research

### 4.1. Failure Analysis of IGBT in Rod Control Power Cabinet

The failure causes of IGBT can be mainly divided into internal failures and external failures. External failures refer to short-term overstress failures caused by extreme external conditions, such as overvoltage, overcurrent, and overheating, during operation. Internal failures refer to the device failure caused by the gradual accumulation of fatigue under the influence of factors such as electrical stress, thermal stress, and mechanical stress [21]. Although external conditions can cause immediate failure, the effects of internal stress and fatigue are more important factors leading to aging failure in the long-term operation of IGBTs.

The rod control power cabinet operates in a complex working environment with changeable working conditions, and the IGBT needs to be switched frequently. The current and voltage flowing through it will have a cumulative impact on the device and eventually lead to the aging failure of the IGBT in advance when it does not reach the design life period. Due to the relatively long life cycle of the IGBT module in practical applications, it is difficult to collect enough degradation process data for fault prediction in a short time. The multi-physical finite element simulation model is used to quickly obtain the total degradation data of the product, analyze the failure principle, and predict the product under normal use conditions according to the aging law under high stress conditions [22,23], which is a method often used in data-driven fault prediction research. It is worth noting that the rod control power cabinet is placed in the electrical building of the nuclear power plant; hence, we do not have to consider the problem of radiation aging. At the same time, because its printed circuit board has been treated with three defenses (moisture, salt spray, and mold), the influence of humidity, salt spray, and mold can also be ignored, and the influence factors such as electrical stress, thermal stress, and mechanical stress are the main causes of concern.

### 4.2. IGBT Modeling and Simulation Based on Multi-Physics Coupling

**Figure 4:**
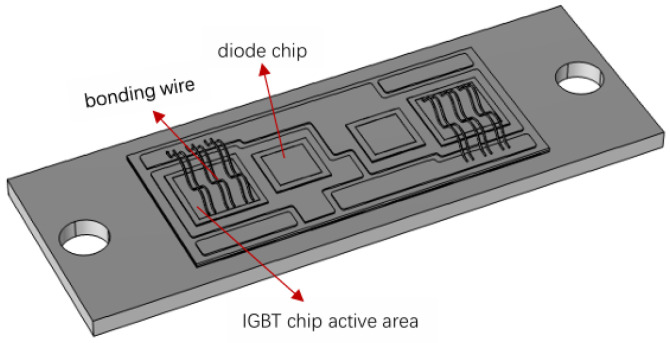
Geometric model of IGBT device.

IGBT devices can be divided into weldability and crimp types according to their package type. At present, the IGBT devices used in the rod control power cabinet of nuclear power plants are weldable-type. In this paper, the 1200 V/75 A IGBT device (2MBI75VA-120-50, manufacturer name Fuji Electric Global, from Tokyo, Japan.) used in the rod control power cabinet of a nuclear power plant is selected as the research object, and COMSOL6.2 software is used to establish the electric-thermo-mechanical coupling finite element simulation model of the IGBT device. Its basic structure is shown in Figure 4.

In the current field, when the IGBT device inputs current, the collector and emitter respectively flow in and out of the current signal, so the collector is set as the end of the current input and the emitter boundary is set to ground. Since the underside of the copper substrate and the heat sink are tightly fitted with thermal silicone grease in the actual operation of the IGBT device, the underside of the copper substrate can be set as a boundary for convective heat flux with a heat transfer coefficient of 5000 W/(m^2^∙K). In addition, since the power loss of the IGBT chip in the working process will produce a certain amount of heat, the IGBT chip is set as the heat source boundary. In the stress field, the bottom surface of the copper substrate and the screw hole are set as fixed constraints, the other parts are regarded as free boundaries, and the initial displacement field is zero.

### 4.3. IGBT Modeling and Simulation Verification

**Figure 5:**
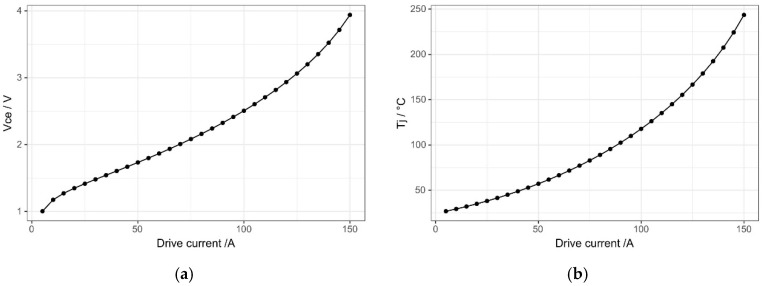
(**a**) Voltage curve of the simulation model under different currents; (**b**) Junction temperature curve of the simulation model under different currents.

Simulation tests were conducted on the 1200 V/75 A IGBT device (2MBI75VA-120-50) using the established IGBT model. The ambient temperature was kept at 25 °C, and the conduction current rose from 0 A to 150 A. The simulation results are shown in Figure 5.

According to the chip reference manual, when the IGBT conduction current is 120 A and the junction temperature is 150 °C, the collector–emitter conduction voltage (Vce) is approximately 2.8 V. Compared with the simulation results, the conduction current is 120 A, the junction temperature is 153 °C, and the Vce is 2.865 V. In the reference manual, when the conduction current is 105 A and the junction temperature is 125 °C, the Vce is approximately 2.6 V. Compared with the simulation results, the conduction current is 105 A, the junction temperature is 126.2 °C, and the Vce is 2.61 V. The simulation results of the above static points are very close to those of the reference manual, which shows the validity of the simulation model results.

In order to further verify the accuracy of the IGBT simulation model, a power cycle experimental platform was established. The current cycle of the chip of the IGBT module was set to be 20 s, the loading cycle was set to be 10 s with an amplitude of 20 A, and then 10 s with an amplitude of 0 A was loaded for the power cycle. The junction temperature of the device was obtained by temperature acquisition card and thermocouple. The results of the junction temperature comparison between the verification experiment and the simulation model are shown in Table 1.

As can be seen from Table 1, the gap between the junction temperature at the start and at the end is relatively large, mainly because there is a delay in the heat conduction between the junction temperature and water-cooled radiator in the actual equipment, and the accumulation and dissipation of heat is not as fast as the ideal situation set by the simulation model, resulting in a large error. Therefore, this part of the data can be excluded. The junction temperatures of the verification experiment and the simulation model are generally consistent, and the error value is small, which further illustrates the validity of the simulation model results.

## 5. IGBT Fault Prediction

### 5.1. Prediction Scheme Design

The operation mode of the welded IGBT device in the rod control power cabinet is closely related to the operation task of the nuclear power plant. These IGBT devices are usually kept in a long-term shutdown state when the reactor is in steady-state operation. When the reactor needs to adjust the power, the rod control power cabinet is required to work continuously. At this time, the IGBT device will undergo the process of switching on, chopping, and switching off periodically according to the control instructions of the reactor power regulation system so as to achieve the rise and fall of the control rod at different speeds. Therefore, the power components of the rod control power cabinet face various operating conditions, including holding conditions and moving conditions. The current and IGBT switching frequencies under typical operating conditions of the rod control power cabinet studied in this paper are shown in Table 2, where the duration of the six states of equivalent current in one cycle under the working conditions is equal.

In the actual work, the holding condition and the movement condition are constantly switched. Generally, it is in the holding condition for a long time. If it is necessary to run, the movement condition lasts for a short time. Based on Table 2, four simulation conditions are preset according to the possible operation requirements of the rod control power cabinet, as shown in Table 3.

The simulation process is described as follows: A 1200 V/75 A IGBT device (model 2MBI75VA-120-50) is selected as the research object, and a multi-physics model is established in COMSOL 6.2 software. Simulations are carried out according to four preset operating conditions. When the device shows a latch-up effect [24], the temperature continues to rise and the thermal runaway phenomenon occurs, the simulation calculation is terminated, and the IGBT device is determined to have a complete aging failure. During the simulation, the characteristic parameters of shell temperature Tc, chip junction temperature Tj, and collector–emitter on-voltage Vce of IGBT devices are monitored and sampled.

With the deepening of the aging degree and performance decline of IGBT devices, their characteristic parameters gradually offset, and after the initial aging occurs and lasts for a period of time, the device undergoes complete aging failure. According to the existing research results, the IGBT module collector–emitter on-voltage Vce is selected as the failure precursor parameter reflecting the aging state of the module, and the Vce is increased by 5% as the failure criterion of the module [25,26]. The simulation results of operating conditions are shown in Table 4. Taking operating condition 1 as an example, after IGBT runs for 15024.5 h continuously under operating condition 1, Vce increases by 5% and the device fails.

In order to fully verify the predictive performance of the proposed method under the combination of multi-source domain conditions, four verification schemes have been designed, as shown in Table 5. In Scheme 1 and Scheme 2, working conditions 3 and 4 with the same conduction current are trained as source domains, and working conditions 1 and 2 are respectively used as target domains for prediction and verification. Scheme 3 and Scheme 4 take condition 1 and condition 2 with the same conduction current as the source domain for training and condition 3 and condition 4 as the target domain for prediction and verification, respectively.

### 5.2. Data Processing

**Figure 6:**
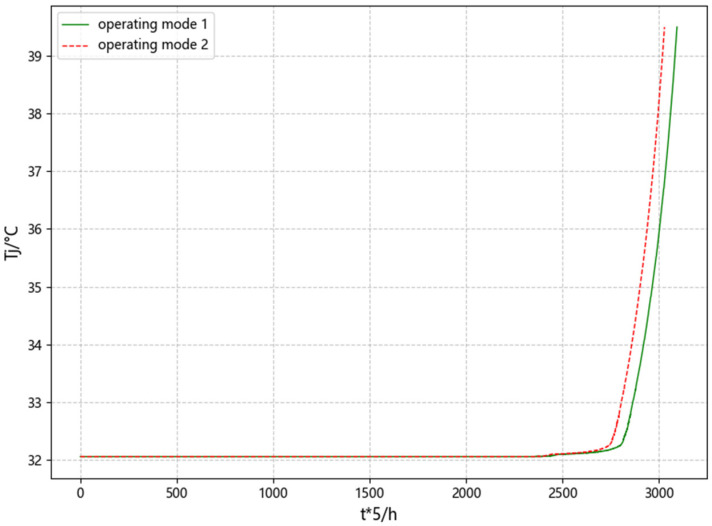
The variation process of junction temperature in operating conditions 1 and 2.

IGBT modules, similar to bearings and mechanical components, usually maintain a stable state of health for a certain period of time [27]. Taking the junction temperature during the long-term operation of operating conditions 1 and 2 as an example, the temperature variation curve is shown in Figure 6. According to the simulation data, in more than half of the simulation process, there is no sign of recession, so it is impossible to predict the failure time from the initial moment. In order to improve the data processing efficiency, optimize data quality, and improve the model generalization ability, all feature parameters are intercepted from 70% of the whole simulation process.

Three parameters, Tc, Tj, and Vce, are selected as characteristic values to reflect the signal change process. By using the KPCA technology, the data of the device shell temperature, chip junction temperature, and collector–emitter on-voltage in multiple source domains are fused to extract three characteristic values to characterize the degradation state of IGBT. On the source domain, 80% of the sample data are selected as the training set of the model, and 20% of the sample data are selected as the model test set. The target domain data are standardized, 70% of the sample data are selected as the training set of the model, and 30% of the data are selected as the model test set.

### 5.3. Results Analysis and Comparison

**Figure 7:**
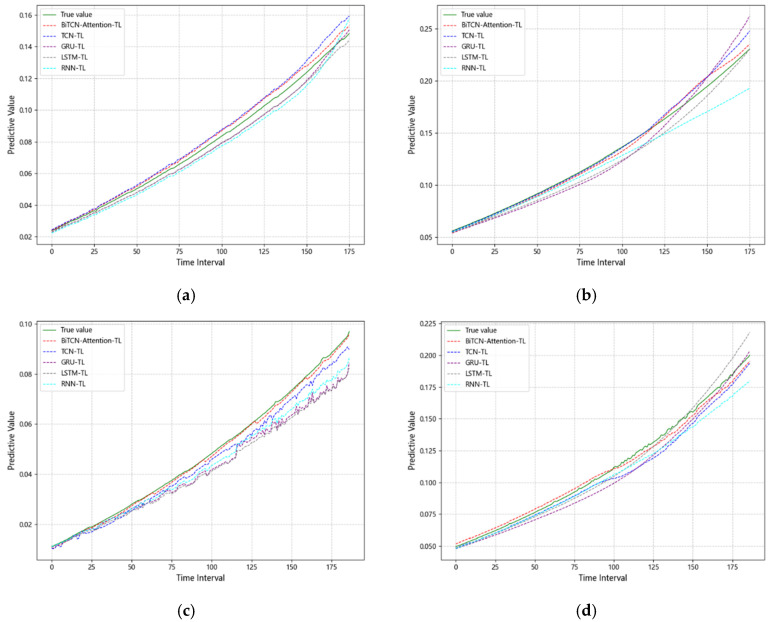
(**a**) Comparison of prediction methods in Scheme 1; (**b**) Comparison of prediction methods in Scheme 2; (**c**) Comparison of prediction methods in Scheme 3; (**d**) Comparison of prediction methods in Scheme 4.

In order to verify the superiority of the IGBT aging fault prediction method proposed in this paper based on the BiTCN-attention transfer learning model, the same data set is used to predict faults by using the RNN-TL model based on recurrent neural networks, the LSTM-TL model based on long short-term memory, the GRU-TL model, and the TCN-TL model. The prediction results of the above five prediction methods on the test set are shown in Figure 7a–d. The mean square error (MSE), root mean square error (RMSE), mean absolute error (MAE), and coefficient of determination (R^2^) are selected as evaluation indexes to analyze the prediction results. The prediction performance evaluation results of the above five prediction methods in Scheme 1, Scheme 2, Scheme 3, and Scheme 4 are shown in Table 6.

As can be seen from the analysis in Table 6 and Figure 7a–d, the BiTCN-attention-TL prediction model proposed in this paper has a higher degree of fitting of prediction curves on the test set compared with the RNN-TL prediction model, GRU-TL prediction model, LSTM-TL prediction model, and TCN-TL prediction model. The prediction errors of MSE, RMSE, and MAE for the BiTCN-attention-TL prediction model are smaller. It is proved that the BiTCN-attention-TL prediction model proposed in this paper has better prediction accuracy and generalization ability and can realize the fault state trend prediction of IGBT more accurately.

## 6. Conclusions

In order to realize the fault prediction of the IGBT power device in nuclear power plants, a transfer learning model based on the BiTCN-attention model is proposed in this paper. By using multi-physics coupling simulation software to model IGBT and verify its accuracy, the IGBT degradation process is simulated by the simulation model, and the IGBT degradation data set under different working conditions is obtained. The KPCA technology is used to integrate the available knowledge of all source domains to predict the target conditions to fully characterize the IGBT degradation state and reduce the computational complexity. Since the BiTCN-attention transfer learning model can consider both past and future information, fully extract important features from time series data through the attention mechanism, and assign different attention weights to input information at different moments, it can more comprehensively capture the long-term dependencies and short-term dynamic changes in time series data. Thus, accurate prediction of cross-operating conditions from source domain to target domain is realized. The results show that the transfer model method can significantly reduce the prediction error, further confirming the excellent prediction performance of the model and filling the gap of existing methods in cross-condition fault prediction. They provide new ideas and technical support for the fault prediction research of the power device IGBT in the rod control cabinet in complex operating environments. Meanwhile, further exploration of various types of source domain data, fully utilizing the IGBT performance data under different operating conditions, should be carried out in the future; this would enhance the diversity and generalization ability of the model and improve its adaptability in a wider range of application scenarios.

## Figures and Tables

**Table 1:** Comparison of Junction Temperature Between Verification Experiment and Simulation Model.

Time/s	0	2	4	6	8	10	12	14	16	18	20
Validation experiment/°C	25.40	29.52	31.30	32.91	34.25	34.47	29.42	27.17	26.50	25.80	25.54
Simulation model/°C	25.09	33.00	34.01	34.51	34.77	34.87	26.90	25.95	25.48	25.27	25.92
Relative error	−1.22%	11.79%	8.66%	4.86%	1.52%	1.16%	−8.57%	−4.49%	−3.85%	−2.05%	1.49%

**Table 2:** Current and IGBT switching frequency under typical operating conditions.

Operating Mode		Switch Status Frequency	Equivalent Current
Holding condition	①	Keep open	15 A
	②	Keep open	10 A
Motion condition 1	①	12 Hz	15 A–30 A–15 A–0 A–0 A–0 A
	②	12 Hz	10 A–20 A–10 A–0 A–0 A–0 A
Motion condition 2	①	6 Hz	22.5 A–45 A–22.5 A–0 A–0 A–0 A
	②	6 Hz	15 A–30 A–15 A–0 A–0 A–0 A
Motion condition 3	①	3 Hz	30 A–60 A–30 A–0 A–0 A–0 A
	②	3 Hz	20 A–40 A–20 A–0 A–0 A–0 A

**Table 3:** Pre-set operating condition table.

Experimental Operating Condition Name	Motion Process
Operating mode 1	No current—Holding condition ① (1 day)—Motion condition 3 ① (20 min)—Motion condition 2 ① (10 min)—Motion condition 1 ① (10 min)—Holding condition ① (1 day)—Motion condition 3 ① (20 min)—Motion condition 2 ① (10 min)—Motion condition 1 ① (10 min)—and so on, until degradation.
Operating mode 2	No current—Holding condition ① (1 day)—Motion condition 1 ① (5 min)—Motion condition 2 ① (15 min)—Motion condition 3 ① (20 min)—Holding condition ① (1 day)—Motion condition 1 ① (5 min)—Motion condition 2 ① (15 min)—Motion condition 3 ① (20 min)—and so on, until degradation.
Operating mode 3	No current—Holding condition ② (1 day)—Motion condition 3 ② (20 min)—Motion condition 2 ② (10 min)—Motion condition 1 ② (10 min)—Holding condition ② (1 day)—Motion condition 3 ② (20 min)—Motion condition 2 ② (10 min)—Motion condition 1 ② (10 min)—and so on, until degradation.
Operating mode 4	No current—Holding condition ② (1 day)—Motion condition 1 ② (5 min)—Motion condition 2 ② (15 min)—Motion condition 3 ② (20 min)—Holding condition ② (1 day)—Motion condition 1 ② (5 min)—Motion condition 2 ② (15 min)—Motion condition 3 ② (20 min)—and so on, until degradation.

**Table 4:** Simulation results of operating conditions.

Experimental Operating Condition Name	Operating Mode 1	Operating Mode 2	Operating Mode 3	Operating Mode 4
Runtime/Hours	15,024.5	14,680.9	56,630.2	54,633.9

**Table 5:** Scheme setting.

Category	Scheme 1	Scheme 2	Scheme 3	Scheme 4
Source domain	Operating mode 3, Operating mode 4	Operating mode 3, Operating mode 4	Operating mode 1, Operating mode 2	Operating mode 1, Operating mode 2
Target domain	Operating mode 1	Operating mode 2	Operating mode 3	Operating mode 4

**Table 6:** Comparison of prediction performance of different methods.

Scheme	Prediction Method	MSE	RMSE	MAE	R^2^
1	RNN-TL	3.13 × 10^−5^	0.00560	0.00512	97.56%
	LSTM-TL	1.48 × 10^−5^	0.00385	0.00354	98.85%
	GRU-TL	1.32 × 10^−5^	0.00363	0.00323	98.97%
	TCN-TL	2.53 × 10^−5^	0.00504	0.00416	98.03%
	BiTCN-attention-TL	1.34 × 10^−6^	0.00116	0.00086	99.89%
2	RNN-TL	2.17 × 10^−4^	0.01475	0.01062	91.32%
	LSTM-TL	7.76 × 10^−5^	0.00881	0.00786	96.91%
	GRU-TL	1.27 × 10^−4^	0.01130	0.00951	94.91%
	TCN-TL	3.24 × 10^−5^	0.00569	0.00381	98.71%
	BiTCN-attention-TL	1.72 × 10^−5^	0.00415	0.00346	99.31%
3	RNN-TL	3.09 × 10^−5^	0.00556	0.00449	94.77%
	LSTM-TL	6.25 × 10^−5^	0.00791	0.00655	89.43%
	GRU-TL	5.29 × 10^−5^	0.00727	0.00575	91.07%
	TCN-TL	9.74 × 10^−6^	0.00312	0.00287	98.35%
	BiTCN-attention-TL	7.49 × 10^−7^	0.00086	0.00076	99.87%
4	RNN-TL	7.72 × 10^−5^	0.00878	0.00694	95.81%
	LSTM-TL	3.48 × 10^−5^	0.00590	0.00475	98.11%
	GRU-TL	5.67 × 10^−5^	0.00752	0.00663	96.93%
	TCN-TL	5.35 × 10^−5^	0.00731	0.00606	97.10%
	BiTCN-attention-TL	1.05 × 10^−5^	0.00325	0.00303	99.42%

## Data Availability

The original contributions presented in the study are included in the article, further inquiries can be directed to the corresponding author.
